# Peptide arrays incubated with three collections of human sera from patients infected with mosquito-borne viruses

**DOI:** 10.12688/f1000research.20981.3

**Published:** 2020-02-28

**Authors:** Maria del Pilar Martinez Viedma, Nurgun Kose, Leda Parham, Angel Balmaseda, Guillermina Kuan, Ivette Lorenzana, Eva Harris, James E. Crowe, Brett E. Pickett

**Affiliations:** 1 J. Craig Venter Institute, La Jolla, CA, 92137, USA; 2 Vanderbilt Vaccine Center, Vanderbilt University Medical Center, Nashville, TN, 37232, USA; 3 Instituto de Investigacion en Microbiologia, Universidad Nacional Autónoma de Honduras, Tegucigalpa, Honduras; 4 Laboratorio Nacional de Virología, Centro Nacional de Diagnóstico y Referencia, Ministry of Health, Managua, Nicaragua; 5 Centro de Salud Sócrates Flores Vivas, Ministry of Health, Managua, Nicaragua; 6 Division of Infectious Diseases and Vaccinology, School of Public Health, University of California, Berkeley, Berkeley, CA, 94720-3370, USA; 7 Department of Pediatrics, Vanderbilt University Medical Center, Nashville, TN, 37232, USA; 8 Department of Pathology, Microbiology, and Immunology, Vanderbilt University Medical Center, Nashville, TN, 37232, USA; 9 Department of Microbiology and Molecular Biology, Brigham Young University, Provo, UT, 84602, USA

**Keywords:** peptide arrays, mosquito-borne viruses, Zika virus, serodiagnostic, bioinformatics, B-cell epitopes

## Abstract

**Background:** Global outbreaks caused by emerging or re-emerging arthropod-borne viruses (arboviruses) are becoming increasingly more common. These pathogens include the mosquito-borne viruses belonging to the *Flavivirus* and *Alphavirus *genera. These viruses often cause non-specific or asymptomatic infection, which can confound viral prevalence studies. In addition, many acute phase diagnostic tests rely on the detection of viral components such as RNA or antigen. Standard serological tests are often not reliable for diagnosis after seroconversion and convalescence due to cross-reactivity among flaviviruses.

**Methods:** In order to contribute to development efforts for mosquito-borne serodiagnostics, we incubated 137 human sera on individual custom peptide arrays that consisted of over 866 unique peptides in quadruplicate. Our bioinformatics workflow to analyze these data incorporated machine learning, statistics, and B-cell epitope prediction.

**Results:** Here we report the results of our peptide array data analysis, which revealed sets of peptides that have diagnostic potential for detecting past exposure to a subset of the tested human pathogens including Zika virus. These peptides were then confirmed using the well-established ELISA method.

**Conclusions:** These array data, and the resulting peptides can be useful in diverse efforts including the development of new pan-flavivirus antibodies, more accurate epitope mapping, and vaccine development against these viral pathogens.

## Introduction

Zika virus (ZIKV) is an arbovirus within the *Flavivirus* genus and the *Flaviviridae* family. In addition to ZIKV, many other mosquito-borne viruses exist that negatively affect public health, including dengue virus (DENV) and chikungunya virus (CHIKV), among others. ZIKV is primarily transmitted by the bite of infected *Aedes* spp. mosquitoes, with limited instances of sexual transmission also being reported ^1– 4^. The recent worldwide epidemic has demonstrated that ZIKV is a neuropathic virus that is associated with fetal microcephaly and other congenital defects in infected pregnant women, and Guillain-Barré syndrome in adults ^5^. Due to the number of ZIKV infections in recent years and the continued threat of ZIKV re-emerging around the world, there is still an urgent need for rapid and accurate surveillance assays in order to rapidly identify new outbreaks. Distinguishing between infection with multiple co-circulating arboviruses that have similar clinical signs and symptoms makes accurate prevalence calculations and diagnosis extremely difficult—especially after convalescence ^6– 10^.

The sequence similarity at the amino acid level in many flavivirus immunogenic protein regions contributes to the observed cross-reactivity in serological assays, which is especially high in the E protein and also present in the NS1 protein ^11^. Although reports showing antibodies against other viral proteins are detectable and show acceptable specificity ^12^, the E and NS1 proteins are considered some of the primary targets of the humoral anti-flavivirus immune response in humans ^13– 15^, even with the known amounts of cross-reactivity with other mosquito-borne flaviviruses.

Recent efforts to generate whole-genome sequences for these pathogens enable the application of bioinformatics tools to mine the data for trends and patterns that can be clinically applicable ^16– 20^. The meta-CATS (metadata-driven Comparative Analysis Tool for Sequences) algorithm is a statistical workflow that rapidly identifies sequence variations that significantly correlate with the associated metadata for two or more groups of sequences ^21^. This algorithm has been included in an analytical workflow to identify residues within 15-mer surface-exposed linear peptide regions that have high predicted specificity and sensitivity values for many Flavivirus species ^22^. The peptides predicted by this prior *in silico* analysis are evaluated in the current study for their ability to detect antibodies against a variety of mosquito-borne viruses. Quantifying the reactivity of this set of peptides using high-throughput custom peptide arrays enables the efficient and simultaneous testing of the set of peptides against a variety of serum samples with higher efficiency than what is possible with manual enzyme-linked immunosorbent assay (ELISA) technology alone ^23^.

The aim of current study is to evaluate the previously-predicted 15-mer viral peptides for their ability to act as differentiating B-cell epitopes, through high-throughput peptide arrays using relevant sera. We have recently completed an analysis of 137 serum samples using custom peptide arrays (each containing 866 experimental viral peptides) to identify 15-mer linear peptides that could be useful as serodiagnostic reagents to detect prior infection with mosquito-borne viruses. Specifically, we tested peptides representing different co-circulating mosquito-borne viruses, including: ZIKV, DENV 1–3, CHIKV and West Nile virus (WNV). Applying machine learning, a weighting scheme, and B-cell epitope prediction algorithms to these data enabled us to identify pools of 8–10 peptides that are predicted to be immunodominant across human sera from previously infected individuals in Central and South America. In addition, we have separately evaluated these peptides using an ELISA method with a set of well-characterized sera. These data could be used by the scientific community to develop improved serological diagnostic methods for detecting past infection with one or more of these viral pathogens.

## Methods

### Peptide preparation and microarray printing

A subset of the previously predicted diagnostic peptides ^22^, representing multiple mosquito-borne virus species and subtypes, were synthesized at the Center for Protein and Nucleic Acid Research at The Scripps Research Institute (TSRI) ^23, 24^. This selected collection of peptides consisted of surface-exposed 15-mers with sequences that represented the consensus amino acid sequence among strains belonging to each of our six target taxa including: CHIKV, DENV1, DENV2, DENV3, WNV, and ZIKV. Peptides on the array that represented mosquito-borne virus taxa for which there were no serum samples were ignored in downstream quantification and computation. As such, a total of 25, 51, 28, 34, or 70 peptides in the E protein as well as 15, 19, 15, 23, or 70 peptides in the NS1 protein (all derived from DENV1, DENV2, DENV3, WNV, or ZIKV sequences, respectively) were evaluated in these experiments. A set of 25 peptides spanning portions of the CHIKV E2 protein that had previously been reported as relevant for detecting anti-CHIKV antibodies were also included ^25^. Synthesized peptides were suspended in 12.5 μL DMSO and 12.5 μL of ultra-pure water. Immediately prior to printing, suspended peptides were diluted 1:4 in a custom protein printing buffer [saline sodium citrate (SSC): 300 mM sodium citrate, pH 8.0, containing 1 M sodium chloride and supplemented with 0.1% Polyvinyl Alcohol (PVA) and 0.05% Tween 20], in a 384-well non-binding polystyrene assay plate. Two positive control peptides, hemagglutinin A (HA) (YPYDVPDYA) and FLAG tag (DYKDDDDK), together with a dye that permanently fluoresces at 488 nm (Alexa Fluor 488) were included in the print to guide proper grid placement and peptide alignment, as well as to serve as printing controls as well as controls to quantify the maximum fluorescence for the assays.

Quadruplicate sets of all peptides were printed onto N-hydroxysuccinimide ester (NHS-ester) coated NEXTERION Slide H (Applied Microarrays) slides at an approximate density of 1 ng/spot, using a Microgrid II (DigilabGlobal) microarray printing robot equipped with solid steel (SMP4, TeleChem) microarray pins. Humidity was maintained at 50% during the printing process. Immediately prior to interrogating the arrays, slides were blocked for 1 h with ethanolamine buffer to quench any unreacted NHS-ester on the slide. All slides were used within 2 months of printing and were stored at -20°C ^23^.

### Serum sources

Spent diagnostic serum samples were provided by collaborators working under three separate clinical studies in Honduras, the United States, and Nicaragua. These sera were collected from a total of 137 consented human patients under IRB supervision and were characterized as positive for antibodies against at least one of: ZIKV, DENV1, DENV2, DENV3, WNV, and/or CHIKV.

A total of 32 deidentified plasma samples from patients suspected of Zika, chikungunya or dengue in Honduras were obtained at the discretion of health care providers at the Hospital Escuela Universitario from patients (ages 6–73 years old). These acute-phase samples were sent to the Centro de Investigaciones Geneticas at the Universidad Nacional Autonoma de Honduras in Tegucigalpa, Honduras for ZIKV, CHIKV and/or DENV molecular testing. Of these patients, 23 had infection with DENV and nine had infection with ZIKV confirmed by RT-qPCR during the acute phase. Convalescent samples were collected from these patients 10–30 days post-onset of symptoms between June 1 to November 30, 2016.

A total of 73 de-identified human serum samples were obtained from the Vanderbilt Vaccine Center Biorepository. Sera from individuals with previous history of natural infection with DENV, WNV, CHIKV, or ZIKV (confirmed by serology for convalescent samples or RT-qPCR for acute-phase samples) while traveling in the Caribbean, Central or South America, or West Africa were included on arrays. For WNV, sera were from individuals with confirmed previous history of natural infection contracted during an outbreak in 2012 in Dallas, TX. The samples were collected in the convalescent phase, months to years after post-onset of symptoms.

A total of 32 de-identified human sera were collected from the Pediatric Dengue Cohort Study (PDCS) in Managua, Nicaragua ^26, 27^. Early convalescent-phase samples were collected 15–17 days post-onset of symptoms from 9 Zika cases that were confirmed as positive for ZIKV infection by real-time RT-qPCR between January and July, 2016. Late convalescent samples were obtained from 21 DENV-positive cohort participants after RT-qPCR confirmed DENV1 (n=7), DENV2 (n=8), or DENV3 (n=6) infection and 2 DENV-negative subjects, all in 2004–2011, prior to the introduction of ZIKV to Nicaragua. Samples were analyzed by inhibition ELISA ^28, 29^ and neutralization assay ^30, 31^. The PDCS was approved by the IRBs of the University of California, Berkeley, and Nicaraguan Ministry of Health. Parents or legal guardians of all subjects provided written informed consent; subjects 6 years old and older provided assent.

### High-throughput screening and quantification of characterized patient sera

Once the peptide microarrays were printed, aliquots from a subset of samples were used to optimize the screening and detection processes. Specifically, dilutions ranging from 1:50 to 1:1000 were evaluated to determine the optimal dilution level for subsequent screening. A 1:200 dilution was selected to achieve an optimal balance between the available aliquot volumes and assay sensitivity.

The 137 characterized sera were separately subjected to high-throughput screening using the synthesized peptide arrays. Sera were tested for IgG reactivity using the custom peptide array at TSRI. For immunolabeling, the incubation area around the printed grids was circumscribed using a peroxidase anti-peroxidase (PAP) hydrophobic marker pen (Research Products International Corp) and the subsequent steps were performed in a humidified chamber at room temperature on a rotator. Control anti-HA (mAb 12CA5, Scripps Research, mouse IgG, RRID:AB_514505) and anti-FLAG monoclonal antibodies (Invitrogen, MA1-142-A488, RRID:AB_2610653) were assayed at a concentration of 10 μg/ml while 10 μl of human sera were diluted 1:200 in PBS buffer containing Tween (PBS-T) and incubated for 1 h followed by three washes in PBS buffer. The arrays were then incubated for 1 h with goat anti-human IgG tagged with Alexa Fluor® 488 (Invitrogen, cat. #: A-11013, RRID: AB_2534080) as a secondary antibody for anti-FLAG and anti-HA bound to control peptides, and Alexa Fluor® 633-conjugated goat anti-human IgG (Invitrogen, cat. #: A-21091, RRID: AB_2535747) as a secondary antibody for serum antibodies bound to viral peptides. Arrays were washed three times in PBS-T, two times in PBS, and another two times in deionized water and centrifuged to dry at 200 × g for 5 mins.

The fluorescence of the processed slides was quantified using a ProScanArray HT (Perkin Elmer) microarray scanner at 488 nm and 635 nm, with laser power set at 5 or 10, PMT gain set at 50 and 50. Captured images were saved as high-resolution TIF files. Imagene® 6.1 microarray analysis software (BioDiscovery; ImageJ could be used as an open-access alternative) was used to calculate the fluorescence intensity of the area within the printed diameter of each peptide as well as the fluorescence of the same diameter directly outside of the area occupied by each peptide. The mean and median fluorescence signal and background pixel intensities, as well as other data for each antigen, spot were calculated, digitized, and exported as individual rows in a comma-delimited file for subsequent analysis.

### Data processing to identify immunodominant epitopes

A custom script ^32^ was written to implement a previously described array processing workflow ^24^ with a minor change to use the median foreground and background values instead of mean values to minimize outlier effects (available on GitHub). Negative background values were interpreted as zeroes. Briefly, background correction was calculated by subtracting the median background from the median foreground measurements for each spot on each array. Normalization was performed by dividing the background-corrected values for each spot on each slide by the non-control spot having the largest fluorescence value on each slide as has been described previously ^24^. The quadruplicate spots for each peptide on each array were then summarized into a single value by calculating the median value of the quadruplicate spots for each peptide to further reduce the effects of any outliers. The normalized relative fluorescence intensity values for all peptides and all samples were output as a separate file together with summarized quantitative values indicating how well each peptide was recognized by each of the polyclonal serum samples.

A separate script was used to transform all relative fluorescence intensity values for each peptide into Z-scores, and separate tables were constructed to contain the summarized Z-score values for all peptides (as columns) representing each of the viral taxa and all samples (as rows) that were tested with the peptide array. A random forest algorithm ( randomForest version 4.6-12 package in R) was applied to each of these tables in order to identify the peptides that were best able to differentiate between each of the viral taxa. In this case, the number of trees generated in the random forest for each species was 100,000, and the number of variables randomly sampled as candidates at each split was equal to the square root of the number of columns present in each table.

The values representing the mean decrease in Gini index were calculated separately for samples obtained from each of the three collections as well as all possible combinations of two or more collections. These data were then used to identify the top 30 peptides according to their usefulness in identifying the correct virus taxon. The BepiPred algorithm was then used to predict the number of residues that are frequently present in B-cell epitopes, and would therefore contribute to increased affinity and binding by antibodies in downstream assays ^33^. The peptides were then assigned a cumulative rank based on the epitope prediction and Gini values, and the 10 highest-ranking peptides across the E and NS1 proteins for each viral taxon, as well as 8 peptides in the E2 region for CHIKV, were categorized as the most likely to have high immunodominance and therefore be recognized by antibodies in sera collected from previously infected patients in the western hemisphere. Statistical comparisons of quantitative differences between the Gini and normalized fluorescence values for sets of peptides were performed using Student’s t-test.

### Peptide validation using ELISA

Each peptide was synthesized (LifeTein, LLC) and 2 ng of peptide was diluted in 50 μL of ddH2O. Natural human IgG protein (abcam, cat. # ab91102), complement component C1q from human serum (sigma, cat. #: C1740), and labelled secondary antibody (ThermoFisher, cat. #: A18817, RRID: AB_2535594) were used as additional controls. Pools of two peptides were used to coat duplicate wells on a 96-well Immulon 4HBX plate (ThermoFisher, cat. # 3855) and incubated at 4°C overnight. Next, 100 μL of blocking buffer (PBS+5% BSA) was added to each well and incubated for 2 hours at room temperature prior to three washing steps with washing buffer (PBS + 0.05% Tween 20). Human serum was diluted 1:25 in blocking buffer and 50 μL of this solution was added to each well prior to incubation for 2 hours at room temperature. Each plate was then washed four times with washing buffer and 50 μL of HRP-conjugated anti-human IgG antibody (ThermoFisher, cat. #: A18817, RRID: AB_2535594; 0.1 mg/mL diluted 1:20,000) was added to each well, followed by incubation at room temperature for 2 hours. Each plate was then washed four additional times before incubating at room temperature for 30 minutes with 75 μL of TMB substrate (abcam, cat. # ab171523). Then, 75 μL of stop solution (abcam, cat. # ab171529) was added to each well and a BioTek-synergy HT plate reader was used to quantify the fluorescence in each well at 450nm within 15 minutes.

### ELISA data processing

A normalization process was implemented that adjusted fluorescence values in each well based on the control wells included on each ELISA plate ^34^, which enabled the downstream comparison between plates. Briefly, the average background value from all negative control wells (i.e. no bound peptide) was calculated and subtracted from each set of duplicate wells on the plate. The normalized value was calculated by dividing the background-corrected value for each set of duplicate wells by the background-corrected average of the positive control wells (i.e. bound secondary antibody). Wells with normalized values of greater than 2.5, between 1.5 and 2.5, or less than 1.5 were categorized as putative positive, borderline, or negative, respectively, for the target viruses. A downstream quality control method was also implemented to ignore results from ELISA plates that displayed high levels of background, inconsistent signal from multiple control wells, or samples observed to have at least two wells for each taxon with higher than expected signal.

### Human subject approval

All samples evaluated on the peptide arrays and ELISA plates were acquired from patients under informed consent and approved by the Ethical or Institutional Review Board at each participating institution, including: Universidad Nacional Autonoma de Honduras (IRB 00003070), Vanderbilt University (IRB 8675), University of California, Berkeley (Committee for Protection of Human Subjects 2010-09-2245), and the Comite Institutional de Revision Etica (NIC-MINSA/CNDR CIRE-09/03/07-008.ver19).

## Results

### Data records

**Figure 1.**
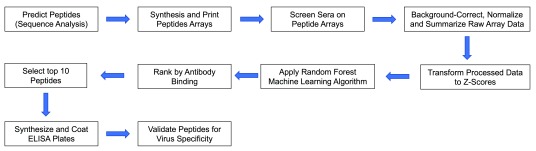
Bioinformatics and laboratory workflow diagram. A graphical depiction of the processes for predicting, screening, processing, and validating peptide array experiments.

Overall, we screened 137 unique serum samples for their reactivity against a panel of viral peptides ( Figure 1 and *Underlying data* ^35^). These samples, together with the clinical diagnosis, were collected from patients with known past exposure to at least one of the viruses targeted by our peptides ( Table 1). Also contained within the *Underlying data* are files describing the metadata of peptides included on the array and each experimental sample ^35^.

**Table 1.** Number of serum samples screened with peptide arrays.

Virus	Number of Samples
CHIKV	5
CHIKV, DENV	32
DENV	10
DENV1	7
DENV2	25
DENV3	12
WNV	12
ZIKV	21
ZIKV, DENV	9
DENV-Negative	2
Unknown	2
Total	137

The data from each array is contained in a single tab-delimited text file and contains the quantitative data captured from a single serum sample on a single peptide array ^35^. A subset of the fields in each file include: location of each peptide spot on the array, peptide identifier, raw mean and median foreground fluorescence at both 488 and 635 nm, raw mean and median background fluorescence at 488 and 635 nm, and other data collected from the raw image.

**Figure 2.**
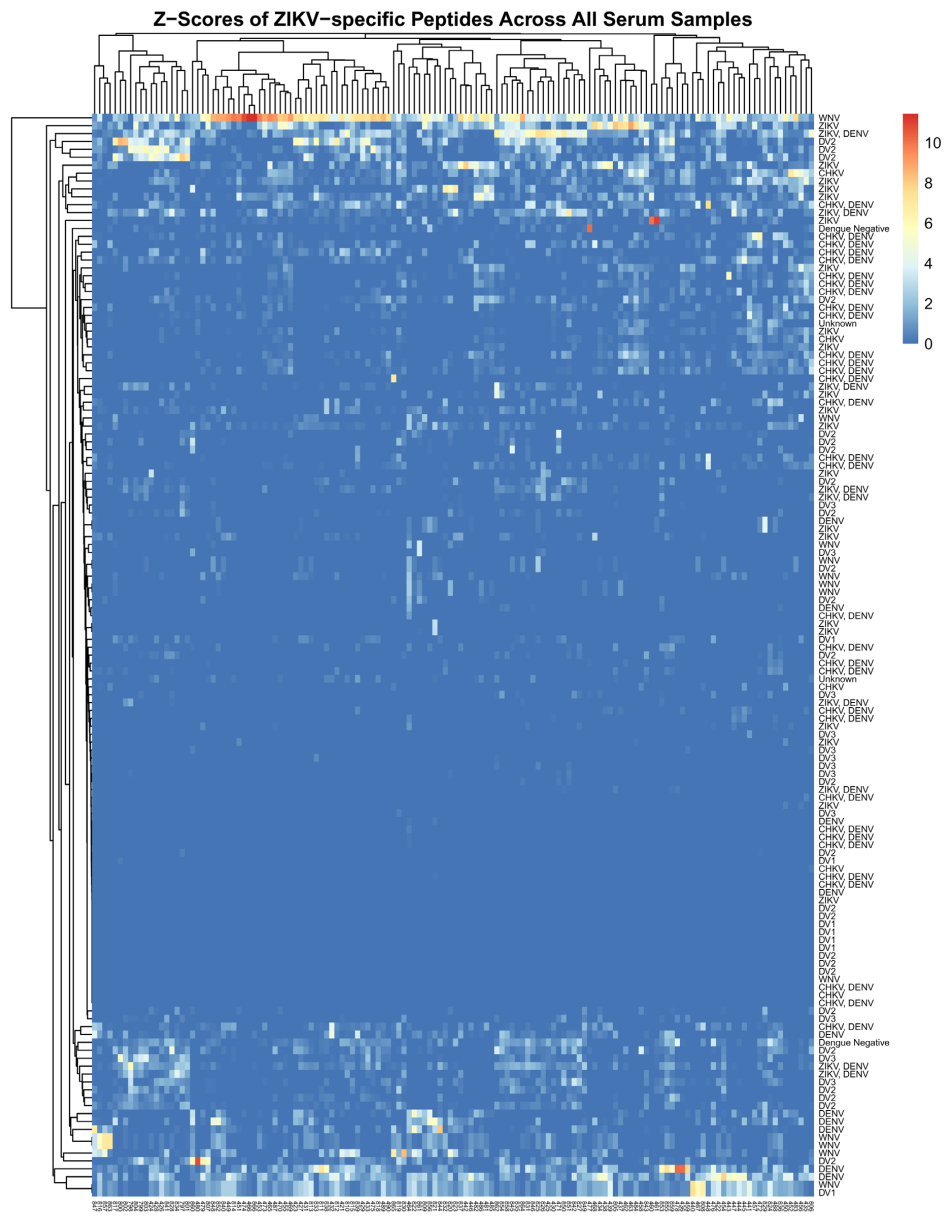
Heatmap of calculated z-score values for ZIKV-specific peptides across all sera. The z-score normalized values (i.e. normal distribution with a mean of 0 and a standard deviation of 1) for all candidate peptides predicted to be specific to ZIKV, together with the known serological history of each patient were visualized in a heatmap with hierarchical clustering.

A subset of Z-scores, which corresponded to peptides predicted to be relevant for ZIKV, were visualized for all samples ( Figure 2). A complete matrix containing the transformed Z-score values for each peptide was formatted for input into a random forest (RF) machine learning algorithm to assist with ranking peptides according to virus taxon. To do so, a column was added to the matrix assigning each sample to the virus taxon that was known to have infected the patient (e.g. “Zika” or “Non-Zika”). Z-score values in columns containing the predicted peptides from each taxon were then captured and input into the RF algorithm.

The benefit of the RF algorithm is that it is capable of ranking the importance of features, which are peptides in this case, based on a known classification. The ranking is based on the mean decrease in Gini index, which is a value that quantifies node impurity. In other words, the higher the Gini index value, the more important the feature is in correctly identifying the virus taxon.

In order to account for geographical, genetic, and population-based factors, we computed the mean decrease in Gini index for individual collections (e.g. Nicaragua or Honduras), all relevant pairs of collections (e.g. Nicaragua and Honduras, Honduras and United States), and the combination of all collections from our sera providers. These calculations were accompanied by a class-error rate that quantifies the number of samples characterized as being positive for ZIKV that were predicted to be ZIKV samples.

This class-error rate information for each individual or combination of collections was then used to weight the peptide rankings results. Briefly, this involved multiplying the average rank for each peptide in each comparison by the average weight and dividing it by the sum of weight. This process works to increase the rank of peptides that have consistently high Gini values. We used these rankings to identify the top 25 species-specific peptides for each virus taxon. This process was repeated for non-ZIKV samples, including WNV, DENV1-3, and CHIKV.

**Figure 3.**
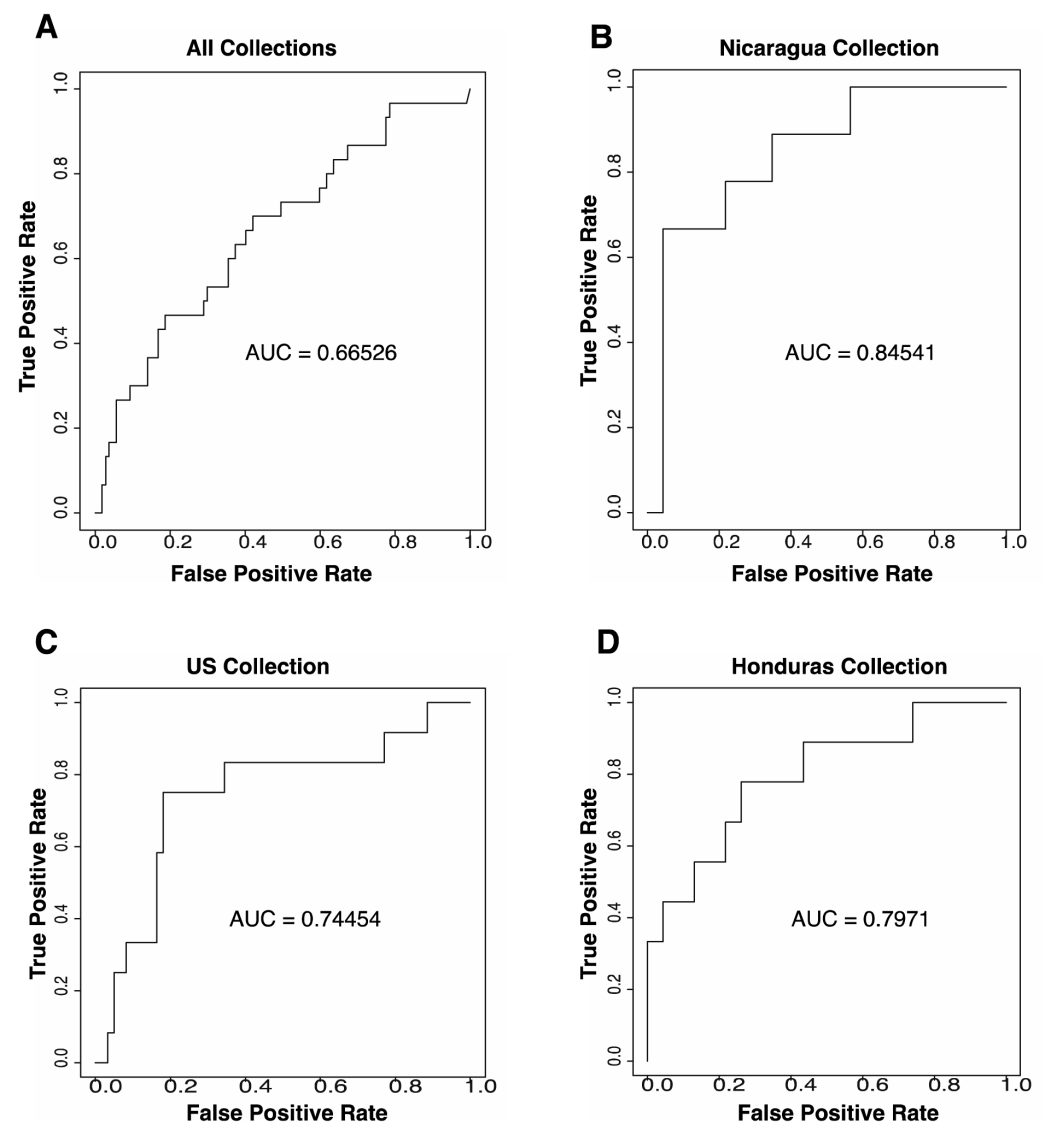
Receiver operating characteristic curve for random forest results. Individual curves were calculated for **A**) the combination all of three collections of sera, **B**) the Nicaragua collection of sera, **C**) the United States collection of sera, and **D**) the Honduras collection of sera.

In addition to the class-error rate, we visualized the random forest output using a receiver operating characteristic (ROC) curve to graph the relationship between true-positive rate and false-positive rate. The area under the curve (AUC) can be calculated with higher values indicating better accuracy. We performed this analysis for the collections from Nicaragua, Honduras, the United States, and the combination of all three collections ( Figure 3).

In order to conserve resources for the peptide array and decrease the number of peptides that would be incorporated into the future ELISA assay, we used the existing BepiPred 2.0 algorithm to predict which of our 15-mer peptides contain the highest number of amino acids that are most often recognized by antibodies ^36^. These B-cell epitope predictions were then used to reduce the 25 best peptides identified from machine learning, to the 10 best peptides that are predicted to not only be species-specific, but that are most likely to contain species-specific epitopes. In the case of ZIKV, we also reviewed the spot size and shape in the peptide array images to ensure that there were no irregularities that could negatively bias our results. The BepiPred 2.0 results enabled us to predict which peptides would be most seroreactive for each selected taxon. The mean maximum score from the BepiPred 2.0 analysis was calculated to be 0.58 (range: 0.55 – 0.63). These scores are associated with a specificity greater than 81%.

### Computational validation

Given the serological cross-reactivity that has been reported among many of our targeted mosquito-borne viruses ^37^, we recognized the need to validate the results of our high-throughput screen. To do so, we not only ensured that those generating the peptide array data were “blinded” to the phenotype of each sample, but we also computationally evaluated two distinct but complementary comparative and quantitative metrics that are described below.

First, we compared two serum samples from pediatric patients that had not been infected with DENV prior to sample collection. The data from the DENV-specific peptides in these samples were then compared to those from a representative DENV-positive sample to verify the differences in signal between known positive and known negative samples. This comparison would also provide a better understanding of the contribution of cross-reactivity, which has been reported previously ^37^, on our platform ( Table 2). This comparison showed that the DENV-negative samples had less than four percent of the normalized fluorescence values, well below the 10 percent that was observed in the DENV-positive sample. Transforming these raw data into Z-scores further increases the observed differences in fluorescence values and, provides additional support to the unbiased nature of the data produced in these experiments.

**Table 2.** Comparison of normalized reactivity percentages among representative well-characterized serum samples as an indicator of peptide specificity.

	DENV-Negative *		DENV-Negative **		DENV1-Positive ***	
	DENV	Non-DENV	DENV	Non-DENV	DENV	Non-DENV
Min	0.055%	0.000%	0.196%	0.392%	0.000%	0.000%
Max	1.496%	7.181%	3.509%	10.201%	10.671%	32.004%
Median	0.383%	0.550%	1.032%	1.854%	1.455%	1.576%
Mean	0.505%	1.005%	1.242%	2.186%	1.837%	3.576%

* Sample H22. ** Sample H23. *** Sample H1.

**Figure 4.**
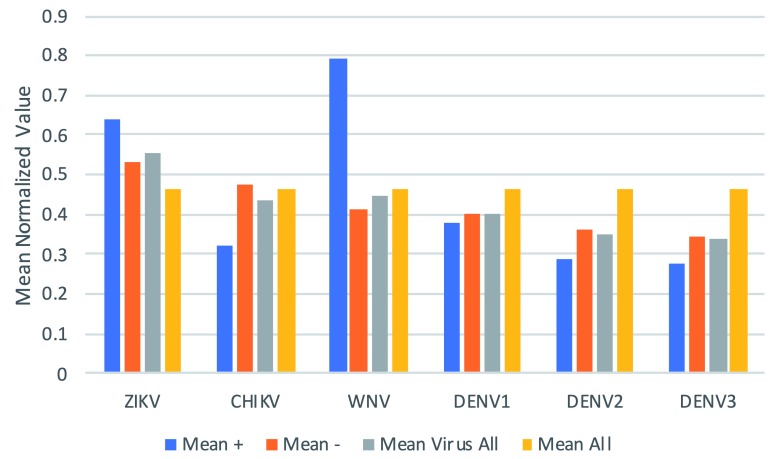
Mean of normalized ratio values across multiple viral taxa. A bar chart depicting the mean values of the best-performing peptides for samples that were characterized as: positive for the specified taxon, negative for the specified taxon, positive or negative for the specified taxon, and all samples across all viral taxa.

**Figure 5.**
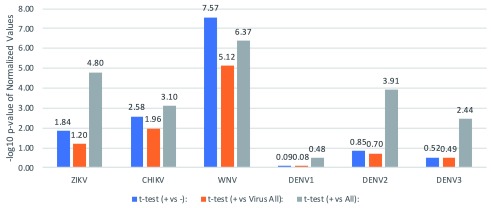
Quantitating statistical significance between normalized ratio values. A bar chart showing the -log10 p-value for the best-performing sets of peptides for each virus species. Comparisons include: samples that were positive for the virus vs. those that were negative for the virus (+ vs -), samples that were positive for the virus vs. all samples quantified for the virus), and samples that were positive vs. all quantified samples (+ vs. All). Values greater than 1.30 indicate p < 0.05.

We next wanted to assess the technical rigor of our approach by performing a statistical analysis of the observed experimental variation in the peptide array experiments. In this case, data was available for six of our target viruses for which sera was evaluated on the arrays. We specifically wanted to quantify the reactivity of the best-performing peptides for each sample against in a panel of comparisons ( Table 3). The results from this analysis identified noticeable differences in the signals for ZIKV and WNV ( Figure 4). However, we observed that the quantified values for the other four virus taxa were lower than the values for all samples combined and did not meet statistical significance when comparing known positive and negative samples ( Figure 5). These results show that incorporating Gini scores and immune epitope predictions into our computational pipeline contributed to our ability to identify sets of peptides that were capable of distinguishing between past infection with a subset of our target viruses.

**Table 3.** Predicted immunodominant diagnostic epitopes identified from peptide array data reported in this study.

ZIKV	CHIKV	WNV	DENV1	DENV2	DENV3
EEWCCRECTMPPLSF	SRKISHSCTHPFHHD	ESCGHRGPATRTTTE	IESEKNETWKLARAS	NIWLKLREKQDVFCD	DLPLPWTSGATTETP
NSFVVDGDTLKECPL	EKFHSRPQHGGKELP	TRMFLKVRESNTTEC	IMWKQISNELNHILL	KEIKVTPQSSITEAE	QKNGSWKLEKASLIE
VREDYSLECDPAVIG	SNAATAEEIEVHMPP	ATVSDLSTKAACPTM	YWIESEKNETWKLAR	KQDVFCDSKLMSAAI	IIGVLEQGKRTLTPQ
AQMAVDMQTLTPVGR	NVYKATRPYLAHCPD	LVHREWFMDLNLPWS	IPFSTQDEKGVTQNG	REKQDVFCDSKLMSA	DGQGKAHNGRLITAN
FVVDGDTLKECPLKH	TDSRKISHSCTHPFH	FVHGPTTVESHGNYS	KCVTKLEGKIVQYEN	TPHSGEEHAVGNDTG	PLPWTSGATTETPTW
GEAYLDKQSDTQYVC	NCKVDQCHAAVTNHK	EWFMDLNLPWSSAGS	FSTQDEKGVTQNGRL	DTGKHGKEIKVTPQS	FSTEDGQGKAHNGRL
GPSLRSTTASGRVIE	IGREKFHSRPQHGGK	DLNLPWSSAGSTVWR	KQISNELNHILLEND	HSGEEHAVGNDTGKH	IGIGDNALKINWYKK
MEIRPRKEPESNLVR	SMGEEPNYQEEWVTH	KAACPTMGEAHNDKR	CKIPFSTQDEKGVTQ	GIMQAGKRSLRPQPT	PWTSGATTETPTWNR
TRGPSLRSTTASGRV		EDFGFGLTSTRMFLK	TDAPCKIPFSTQDEK	HGKEIKVTPQSSITE	CKIPFSTEDGQGKAH
KNDTWRLKRAHLIEM		HGPTTVESHGNYSTQ	DEKGVTQNGRLITAN	GNDTGKHGKEIKVTP	TSGATTETPTWNRKE

It is also important to recognize that each peptide was printed at non-adjacent sites on each array in quadruplicate to minimize experimental bias due to the location of any given spot on the array. Incorporating technical replicates was an important component of the experimental design. Such an approach enables improved replication of the results and also increases the scientific rigor of the resulting dataset upstream of any data processing workflows.

### Experimental validation

**Figure 6.**
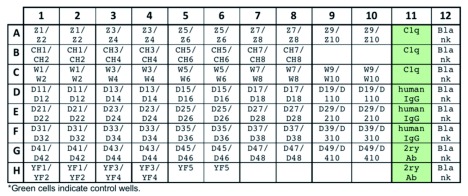
Layout of custom ELISA plate to screen sera for reactivity against predicted peptides. Testing of duplicate pairs of each peptide for each of the target virus taxa. Each column contains pairs of peptides that were predicted to be effective at differentiating each virus based on the bioinformatics processing of the peptide array data. (row A for ZIKV; row B for CHKV; row C for WNV; row D for DENV-1; row E for DENV-2; row F for DENV-3; row G for DENV-4; row H for YFV). Negative control wells, which were not coated with peptides are in column 12 and in wells with no label. Wells containing other control material are in column 11 (green) and include: C1q, naïve human immunoglobulin G, or only secondary antibody.

The number of samples that were evaluated for prior exposure to each virus was insufficient to allow the use of *in silico* cross-validation techniques that are generally applied to the classifier predictions. We therefore designed custom 96-well ELISA plates to validate the ability of the peptides ( Figure 6). The highest predicted reactivity to accurately detect prior infection by each of the target viruses.

 These custom ELISA plates were incubated with 26 human convalescent sera that had been previously characterized as positive for at least one of our target virus taxa using complementary methods, including plaque reduction neutralization test (PRNT) from convalescent serum, IgM antibody capture enzyme-linked immunosorbent assay (MAC-ELISA) from post-acute phase serum, and/or quantitative real-time PCR (qRT-PCR) from blood collected during acute infection. These samples were obtained from public sources including: BEI Resources (5 samples), the World Reference Center for Emerging Viruses and Arboviruses (7 samples), or the United States Centers for Disease Control and Prevention (16 samples).

After processing and correcting the raw ELISA data, we found that the well-characterized samples showing a normalized absorbance ratio greater than 1.5 correlated with cases of previously confirmed Zika infection ( Table 5– Table 30). Consequently, we compiled the normalized absorbance ratio data and categorized any peptide pool found to have a normalized ratio value greater than 1.5 was classified as a “borderline” result, while those having a value greater than 2.5 was classified as a putative “positive” result. In order to increase specificity, any sample with at least two wells of the ELISA plate having normalized ratios greater than 1.5 were labeled as putative “positive” for prior infection with the target virus.

Given the p-values associated with the peptide array results, we decided to especially focus on samples that were positive for ZIKV. As such, instances where excessive signal was detected for all viruses were processed in a way that still identified samples having at least 2x stronger signal for ZIKV peptides than for DENV peptides in the same sample were labeled with a “Z” to differentiate them from other categories.

The summarized results of the ELISA data revealed a fair amount of concordance with the “gold standard” methods and displayed overall sensitivity and specificity of 61.5% and 50%, respectively ( Table 4). Interestingly, these values fluctuated depending on the collection that was analyzed and were affected by small sample size from two of the collections.

**Table 4.** ELISA data compared with sample characterization data and metadata.

Control Sample Name	ZIKV (P-ositive, N-egative, B-orderline, Z-ika adjustment)	ZIKV Conclusion (INCLUDE 'Z')	ZIKV Conclusion (EXCLUDE 'Z')	Interpretation ZIKV	PRNT new guidance interpretation	PRNT 4-fold difference interpretation	MAC- ELISA P/N ZIKV	PRNT ZIKA	PRNT DENV 1	PRNT DENV 2	PRNT DENV 3	PRNT DENV 4	Original Clinical PCR result	Original Clinical IgM result	Original Clinical PRNT interpretation	Time from clin sample to conv sample	Specificity (Include 'Z')	Sensitivity (Include 'Z')	Specificity (All)	Sensitivity (All)
NR50226D2	#N/A	FP	TN	--	--	--	--	--	< 20	271	< 20	42	--	--	Primary DENV2	--	0	0	0.25	0.615
NR50231D	#N/A	FP	FP	--	--	--	--	--	91	293	350	68	--	--	Secondary DENV	--				
NR50896ZK	#N/A	FN	FN	Positive	--	--	--	--	--	--	--	--	--	--	ZIKV	89 days				
NR50900ZK	#N/A	FN	FN	Positive	--	--	--	--	--	--	--	--	--	--	ZIKV	100 days				
NR50902ZK	#N/A	--	--	Positive	--	--	--	--	--	--	--	--	--	--	ZIKV	170 days				
UTMB10	#N/A	--	--	Positive	--	--	3.83	6758	52	42	57	43	--	--	--	97 days	NaN	1		
UTMB11	#N/A	--	--	Positive	--	--	2.82	3542	47	<30	76	48	--	--	--	135 days				
UTMB133	#N/A	TP	FN	Positive	--	--	--	--	--	--	--	--	Positive	--	--	--				
UTMB134	#N/A	TP	FN	Positive	--	--	--	--	--	--	--	--	Positive	--	--	--				
UTMB2	#N/A	TP	TP	Positive	--	--	4.01	3106	2765	1876	2438	1230	--	--	--	45 DPO				
UTMB8	#N/A	--	--	Positive	--	--	1.27	658	ND	ND	ND	ND	--	--	--	113 DPO				
UTMB9	#N/A	--	--	Positive	--	--	1.39	438	376	412	453	425	--	--	--	156 DPO				
P23-A	#N/A	--	--	Presumptive Positive	flavivirus	flavivirus	40.99	2560	10240	5120	--	--	Pos	--	--	^~^ 6–8 weeks	0.5	0.625		
P23-B	#N/A	FN	FN	Presumptive positive	flavivirus	flavivirus	12.03	2560	2560	5120	--	--	Pos	--	--	^~^ 6–8 weeks				
P23-C	#N/A	TP	TP	Presumptive positive	flavivirus	zika	9.44	1280	80	20	--	--	Pos	--	--	^~^ 6–8 weeks				
P23-D	#N/A	--	--	Presumptive positive	flavivirus	dengue	7.91	1280	>20480	2560	--	--	Pos	--	--	^~^ 6–8 weeks				
P23-E	#N/A	FN	FN	Presumptive positive	flavivirus	flavivirus	8.21	1280	1280	1280	--	--	Neg	Neg/Pos seroconversion	flavivirus	4 mos				
P23-F	#N/A	--	--	Presumptive positive	flavivirus	zika	9.79	1280	10	<10	--	--	Pos	IgM Positive	flavivirus	15 weeks				
P23-G1	#N/A	FP	TN	Negative	Negative	Negative	--	--	--	--	--	--	--	--	--	--				
P23-H	#N/A	--	--	Presumptive positive	flavivirus	zika	25.78	320	10	<10	--	--	Pos	IgM Positive	flavivirus	9 weeks				
P23-I	#N/A	TP	TP	Presumptive Positive	flavivirus	flavivirus	4.6	640	5120	10240	--	--	Pos	--	--	^~^ 6–8 weeks				
P23-J1	#N/A	TN	TN	Negative	Negative	Negative	--	--	--	--	--	--	--	--	--	--				
P23-K	#N/A	FN	FN	Presumptive Positive	flavivirus	flavivirus	44.66	1280	1280	1280	--	--	Pos	--	--	^~^ 6–8 weeks				
P23-L	#N/A	TP	FN	Presumptive Positive	flavivirus	flavivirus	55.22	1280	1280	640	--	--	Pos	--	--	^~^ 6–8 weeks				
P23-M	#N/A	TP	FN	Presumptive Positive	flavivirus	zika	14.16	10240	80	40	--	--	Pos	--	--	^~^ 6–8 weeks				
P23-N	#N/A	TP	FN	Presumptive Positive	flavivirus	flavivirus	60.86	2560	5120	1280	--	--	Pos	--	--	^~^ 6–8 weeks				

**Table 5.** Normalized ELISA results for NR50226D2.

	1	2	3	4	5	6	7	8	9	10	11	12
A	1.654		1.038		1.038		3.038		0.692		50.192	
B	0.462		1.346		1.038		2.077		0.346			
C	0.962		2.154		1.346		1.115		0.923			
D	2.346		1.077		2.231		0.885		0.538		122.500	
E	2.385		1.077		0.346		0.692		0.423			
F	1.115		0.500		1.192		1.154		14.308			
G	0.692		2.192		1.577		0.769		2.538		1.000	
H	2.038		3.231		2.077		0.615		0.808			

**Table 6.** Normalized ELISA results for NR50231D.

	1	2	3	4	5	6	7	8	9	10	11	12
A	0.161		1.252		1.671		0.776		1.699		13.531	
B	1.308		1.839		10.958		14.203		0.664			
C	1.196		1.336		0.189		1.503		1.699			
D	1.140		1.392		0.860		1.112		0.776		85.737	
E	1.811		0.720		0.748		0.664		1.112			
F	0.748		1.531		0.972		1.140		1.755			
G	0.636		1.531		1.615		1.671		2.594		1.000	
H	1.364		4.664		2.035		0.217		0.049			

**Table 7.** Normalized ELISA results for NR50896ZK.

	1	2	3	4	5	6	7	8	9	10	11	12
A	0.400		0.560		0.240		0.480		-0.240		75.600	
B	-0.840		1.280		0.120		1.240		-0.320			
C	-0.120		1.480		0.600		0.240		0.400			
D	1.560		0.880		2.760		-0.080		-0.640		122.907	
E	2.440		0.600		0.880		0.120		1.080			
F	0.520		-0.080		0.040		0.080		23.960			
G	0.480		1.640		1.560		0.400		0.360		1.000	
H	1.640		2.320		2.480		0.040		0.080			

**Table 8.** Normalized ELISA results for NR50900ZK.

	1	2	3	4	5	6	7	8	9	10	11	12
A	0.265		-0.306		0.020		-0.959		1.000		35.204	
B	1.000		1.245		-0.306		-0.224		-1.449			
C	25.163		0.102		-1.204		-0.306		0.429			
D	1.000		-0.796		-0.388		-1.776		-1.204		254.796	
E	1.816		-0.796		1.327		-0.061		0.673			
F	0.265		0.265		0.102		0.510		11.694			
G	0.592		0.918		2.224		0.510		3.694		1.000	
H	4.755		28.347		4.102		-0.224		0.347			

**Table 9.** Normalized ELISA results for NR50902ZK.

	1	2	3	4	5	6	7	8	9	10	11	12
A	1.480		0.200		-0.600		-0.440		2.440		86.707	
B	-0.920		1.640		-0.120		-1.240		-3.160			
C	39.720		0.520		-1.080		-0.440		0.520			
D	3.400		-0.440		1.320		-3.320		-0.760		526.173	
E	2.280		-1.400		2.440		0.840		2.760			
F	-0.920		-0.920		0.840		0.840		18.280			
G	0.200		2.440		0.360		-0.280		5.160		1.000	
H	3.560		49.160		2.920		0.040		0.200			

**Table 10.** Normalized ELISA results for P23-A.

	1	2	3	4	5	6	7	8	9	10	11	12
A	2.862		3.655		2.828		2.138		3.586		18.000	
B	4.172		5.379		2.966		3.793		-0.310			
C	3.310		5.897		5.552		4.828		6.379			
D	8.379		6.897		3.345		2.379		3.379		53.080	
E	4.552		3.655		5.103		4.586		2.966			
F	3.655		5.103		5.034		5.655		2.724			
G	4.517		4.000		7.103		4.828		1.103		1.000	
H	3.207		2.483		3.828		-0.241		-0.276			

**Table 11.** Normalized ELISA results for P23-B.

	1	2	3	4	5	6	7	8	9	10	11	12
A	-1.235		-3.431		-4.137		-2.098		-15.510		-1.340	
B	-2.647		-4.529		-3.980		-3.902		1.000			
C	-1.471		-6.529		-3.039		-3.784		-4.059			
D	-6.725		-5.863		-30.647		-3.588		-1.275		-64.634	
E	-4.412		-4.843		-3.667		-5.510		-0.137			
F	-3.902		-4.451		-4.020		-4.647		-4.098			
G	-4.490		-5.157		-5.824		-1.667		0.882		1.000	
H	-1.353		-5.157		-2.961		0.333		1.392			

**Table 12.** Normalized ELISA results for P23-C.

	1	2	3	4	5	6	7	8	9	10	11	12
A	-3.632		0.860		1.842		2.263		6.404		-0.450	
B	-3.211		-0.333		1.491		1.421		-0.825			
C	-5.947		5.421		3.526		3.807		7.316			
D	6.474		3.246		1.211		-3.561		-1.105		94.708	
E	1.561		0.088		2.474		1.070		-1.947			
F	-1.316		1.561		4.649		4.649		9.702			
G	0.649		1.632		6.333		1.912		-1.175		1.000	
H	-0.684		-0.754		3.246		-1.877		0.789			

**Table 13.** Normalized ELISA results for P23-D.

	1	2	3	4	5	6	7	8	9	10	11	12
A	1.216		7.054		4.784		4.459		10.838		18.622	
B	1.324		1.973		-0.405		4.459		-1.054			
C	-3.649		9.649		5.324		7.162		7.703			
D	19.162		6.514		3.919		-5.054		-2.784		171.559	
E	8.459		-1.270		1.757		-0.622		-2.459			
F	5.000		2.730		5.541		4.892		-3.541			
G	2.189		5.000		11.378		2.514		-1.378		1.000	
H	-6.568		1.541		1.865		-6.459		-1.378			

**Table 14.** Normalized ELISA results for P23-E.

	1	2	3	4	5	6	7	8	9	10	11	12
A	-0.051		-0.333		-0.795		0.000		-1.487		-21.829	
B	-0.231		-0.718		0.179		-0.923		0.026			
C	0.641		-0.872		-0.538		-1.385		-1.718			
D	-1.641		-0.667		1.385		1.436		0.051		-53.145	
E	-0.795		0.103		1.051		0.923		-0.487			
F	-0.205		-0.487		-1.051		-1.231		-0.077			
G	-0.282		-0.897		-1.359		-0.154		0.103		1.000	
H	-0.205		0.026		-0.410		0.641		0.872			

**Table 15.** Normalized ELISA results for P23-F.

	1	2	3	4	5	6	7	8	9	10	11	12
A	0.918		-0.061		-3.735		-1.694		-4.061		-10.728	
B	1.082		-1.612		1.245		-3.735		2.959			
C	3.204		-2.265		-1.857		-3.653		-4.633			
D	-1.612		-0.388		3.694		2.143		-1.694		-144.007	
E	-2.673		-0.633		-7.571		1.980		-3.163			
F	1.816		0.510		-2.265		-4.633		1.163			
G	1.408		-1.367		-2.918		1.082		1.490		1.000	
H	2.878		-1.531		1.245		3.531		3.612			

**Table 16.** Normalized ELISA results for P23-G.

	1	2	3	4	5	6	7	8	9	10	11	12
A	-0.679		-1.272		-3.198		-1.123		-5.568		-5.930	
B	-1.469		-3.494		-1.222		-3.593		-0.037			
C	1.000		-6.160		-5.519		-3.148		-5.617			
D	-5.914		-20.136		1.741		0.457		-0.185		-114.111	
E	-12.580		-3.049		0.111		-0.037		-46.753			
F	-1.370		-1.864		-4.185		-4.037		-63.543			
G	-2.259		-3.198		-5.519		-0.877		0.309		1.000	
H	-1.025		1.444		-18.901		5.741		3.222			

**Table 17.** Normalized ELISA results for P23-H.

	1	2	3	4	5	6	7	8	9	10	11	12
A	-1.000		43.000		36.000		68.000		69.000		116.667	
B	21.000		54.000		15.000		60.000		-5.000			
C	-54.000		88.000		35.000		67.000		73.000			
D	105.000		33.000		26.000		-14.000		-38.000		2154.000	
E	24.000		-8.000		6.000		1.000		-5.000			
F	22.000		12.000		46.000		52.000		-24.000			
G	20.000		25.000		99.000		22.000		-23.000		1.000	
H	57.000		100.000		41.000		-24.000		-15.000			

**Table 18.** Normalized ELISA results for P23-I.

	1	2	3	4	5	6	7	8	9	10	11	12
A	-3.000		-1.000		0.455		0.273		4.455		7.273	
B	-1.545		0.182		-2.727		1.909		-3.273			
C	-3.455		1.455		-3.091		2.727		5.091			
D	2.545		-0.727		-7.273		-7.818		5.818		118.909	
E	2.455		-3.273		-4.364		-3.727		-4.091			
F	-1.636		0.636		-1.182		0.909		-5.909			
G	-1.091		-1.273		2.273		-0.818		1.364		1.000	
H	0.273		-3.364		0.091		-5.455		-15.545			

**Table 19.** Normalized ELISA results for P23-J.

	1	2	3	4	5	6	7	8	9	10	11	12
A	-2.067		-18.733		-21.400		-23.400		-31.000		16.422	
B	-20.067		-25.933		-18.600		-28.067		0.067			
C	-1.533		-30.333		-19.400		-28.467		-42.733			
D	-44.333		-26.867		4.600		7.400		-17.667		-243.578	
E	-38.067		-12.067		-9.267		-13.933		-7.800			
F	-23.000		-26.200		-17.133		-27.133		-4.067			
G	-23.933		-21.400		-35.800		-17.800		-1.667		1.000	
H	-21.133		-19.267		-22.867		3.400		8.067			

**Table 20.** Normalized ELISA results for P23-K.

	1	2	3	4	5	6	7	8	9	10	11	12
A	-1.900		-2.000		0.500		0.900		-0.100		-0.133	
B	-3.200		0.000		-2.100		2.600		-1.900			
C	-3.500		3.400		-0.300		3.400		5.600			
D	2.700		-2.800		-7.300		-1.000		7.900		171.533	
E	1.100		-3.100		-2.700		-2.400		-2.500			
F	-0.500		2.100		1.200		3.400		0.200			
G	3.000		1.600		6.500		2.000		0.700		1.000	
H	-0.400		1.600		3.500		-1.000		-2.600			

**Table 21.** Normalized ELISA results for P23-L.

	1	2	3	4	5	6	7	8	9	10	11	12
A	-2.647		1.588		1.706		1.471		7.353		5.118	
B	3.471		1.471		-0.059		1.706		-1.000			
C	-6.765		3.235		1.706		7.118		11.000			
D	1.941		-0.294		-8.294		-12.176		4.765		152.647	
E	4.647		-7.353		-3.941		-3.941		-2.294			
F	2.059		3.235		2.059		4.647		-5.941			
G	1.706		3.706		6.294		0.882		-0.176		1.000	
H	5.588		0.059		2.529		-3.118		-1.941			

**Table 22.** Normalized ELISA results for P23-M.

	1	2	3	4	5	6	7	8	9	10	11	12
A	-0.667		-1.500		-2.250		-0.667		-3.750		-20.278	
B	0.000		-1.333		-0.250		-2.417		0.167			
C	-1.250		-3.500		-2.000		-3.000		-2.167			
D	-3.333		-0.667		1.333		-0.250		-2.583		-169.667	
E	-2.750		-2.000		0.250		-0.500		0.083			
F	0.250		0.167		-2.500		-1.667		-4.917			
G	-0.167		-2.000		-1.667		0.333		0.083		1.000	
H	4.333		1.500		2.667		4.333		5.000			

**Table 23.** Normalized ELISA results for P23-N.

	1	2	3	4	5	6	7	8	9	10	11	12
A	7.526		8.158		9.947		8.579		17.421		64.895	
B	7.737		9.737		10.684		11.737		5.842			
C	6.368		12.158		8.684		12.895		13.632			
D	14.053		7.737		10.789		3.632		6.579		193.105	
E	9.421		6.368		7.105		8.158		9.105			
F	7.947		8.789		11.316		13.000		7.105			
G	11.000		9.632		14.474		9.632		7.947		1.000	
H	3.000		4.053		3.737		-0.263		-0.579			

**Table 24.** Normalized ELISA results for UTMB2.

	1	2	3	4	5	6	7	8	9	10	11	12
A	1.640		1.000		0.787		0.733		2.173		46.351	
B	1.267		0.307		1.213		1.907		-0.067			
C	-1.507		1.267		1.480		0.680		0.573			
D	0.893		-0.493		5.320		-0.333		0.200		160.307	
E	3.507		-0.707		5.320		1.587		2.813			
F	0.307		-0.280		0.467		0.413		13.480			
G	0.200		1.693		1.267		0.147		-0.440		1.000	
H	0.627		4.787		1.000		-0.227		-1.560			

**Table 25.** Normalized ELISA results for UTMB8.

	1	2	3	4	5	6	7	8	9	10	11	12
A	4.155		2.859		1.113		1.451		2.352		90.296	
B	3.648		3.873		3.028		3.648		0.606			
C	1.789		2.296		1.789		3.986		1.620			
D	4.155		1.507		16.155		-0.408		1.451		157.901	
E	5.000		0.606		0.831		1.563		1.563			
F	2.070		1.732		2.915		1.676		1.901			
G	2.183		3.873		2.408		0.887		5.000		1.000	
H	3.141		2.859		1.563		1.225		0.437			

**Table 26.** Normalized ELISA results for UTMB9.

	1	2	3	4	5	6	7	8	9	10	11	12
A	-25.667		-24.333		-36.333		-19.000		-20.333		-566.111	
B	22.333		-111.000		-19.000		-16.333		22.333			
C	2.333		-51.000		-37.667		-29.667		-4.333			
D	-52.333		-33.667		-159.000		-16.333		-15.000		-3714.556	
E	-57.667		-19.000		-27.000		-23.000		-21.667			
F	-33.667		-35.000		-48.333		-132.333		-39.000			
G	-21.667		-61.667		-21.667		-133.667		-33.667		1.000	
H	-9.667		-292.333		-316.333		6.333		-19.000			

**Table 27.** Normalized ELISA results for UTMB10.

	1	2	3	4	5	6	7	8	9	10	11	12
A	14.067		13.800		17.267		3.667		7.400		321.089	
B	2.067		6.333		12.733		15.133		-6.733			
C	6.867		10.333		8.733		3.667		2.333			
D	15.133		9.533		5.800		-3.800		-3.800		697.800	
E	14.600		3.667		-9.933		-0.067		-0.867			
F	6.333		1.800		13.533		47.133		1.267			
G	5.533		5.800		4.733		28.467		14.600		1.000	
H	2.867		14.067		0.200		0.200		2.867			

**Table 28.** Normalized ELISA results for UTMB11.

	1	2	3	4	5	6	7	8	9	10	11	12
A	6.435		7.826		7.478		4.217		3.913		41.101	
B	2.217		45.696		6.174		7.391		-0.478			
C	0.957		3.391		5.783		3.739		3.304			
D	6.391		3.000		10.826		1.391		1.652		121.507	
E	6.870		1.913		1.913		3.739		1.348			
F	0.174		1.174		5.304		2.652		0.913			
G	3.348		5.174		3.957		1.478		9.130		1.000	
H	3.391		7.870		7.304		-1.391		0.174			

**Table 29.** Normalized ELISA results for UTMB133.

	1	2	3	4	5	6	7	8	9	10	11	12
A	4.268		3.479		1.676		1.282		4.549		30.690	
B	3.141		2.634		-0.127		-1.535		-0.915			
C	-0.127		-0.352		0.718		-1.817		3.535			
D	-0.070		2.352		5.620		-3.394		1.394		153.094	
E	9.845		-1.761		3.141		0.042		-0.972			
F	2.014		2.465		3.986		3.366		-0.577			
G	3.873		4.099		4.324		0.718		8.042		1.000	
H	3.254		9.620		-0.915		-3.056		-3.338			

**Table 30.** Normalized ELISA results for UTMB134.

	1	2	3	4	5	6	7	8	9	10	11	12
A	-2.250		-1.500		-1.850		-1.125		-2.150		-10.992	
B	-1.750		-2.050		-0.550		-1.375		0.075			
C	-1.350		-2.550		0.350		-0.950		-0.750			
D	-2.575		-1.025		-3.025		-0.275		0.050		-68.275	
E	-3.075		-1.200		-0.650		-1.225		-0.650			
F	-1.000		-0.625		-0.825		-0.775		-3.400			
G	-2.175		-1.875		-1.425		-0.500		-1.900		1.000	
H	-1.300		-2.150		-1.300		0.000		0.050			

## Discussion

The array data reported in this manuscript were used to identify high-scoring peptides that could be used as serodiagnostic reagents in an ELISA format to distinguish between prior infection and seroconversion to a panel of mosquito-borne viruses. Our workflow incorporated both computational and laboratory components to improve identification of regions that were uniquely recognized by virus-specific antibodies to each virus and could therefore be useful as serodiagnostic peptides. Sabalza *et al.* described a protocol to identify ZIKV specific diagnostic epitopes through peptide microarrays; however, they only used one human serum sample, did not use any bioinformatics analysis, and the identified peptides sequences were not provided ^38^.

The integration of Gini values calculated by the random forest machine learning algorithm with the BepiPred B-cell epitope prediction algorithm, enabled us to identify the best peptides for each taxon. This approach improved our selected peptides to those that had increased affinity and binding to antibodies ^33^. We purposely chose peptides in both the E and NS1 proteins (E2 protein of CHIKV) to improve our ability to detect epitopes within viral antigens that are known to circulate in the bloodstream ^11^. However, it would be of broad interest to quantify how the specificity and sensitivity of our assay would be affected by including additional peptides that span the viral nonstructural protein regions.

We observed that a few of our selected peptides displayed high reactivity and Gini values, while other selected peptides had lower measured values. We attribute a subset of these unexpected differences to the imposed requirement of being located within a predicted B-cell epitope. Is also possible that the heavy + light chain secondary antibodies used for the array could also bind IgM and had an affect the quantified reactivity. Reactivity is an essential measurement for individual samples, while Gini values are useful to rank peptides based on their ability to identify peptides that differentiate one taxon from the others. As such, Gini values are better able to identify linear epitopes that differentiate taxa and that are sufficiently immunodominant across patient populations. We are therefore confident in the results from taxa where the Gini values were significantly different between selected peptides when compared to the remaining peptides. By providing the raw data in a publicly-accessible resource, we expect these data to be subject to re-analysis and meta-analysis using alternative methods.

We also noticed cases where the comparisons of our selected peptides yielded non-significant p-values in various comparisons, especially among dengue viruses. The most likely explanation for this observation is the high degree of cross-reactivity that occurs between linear epitopes derived from these viruses. While other existing serological assays are capable of distinguishing between these highly related taxa, they primarily rely on recognition of conformational epitopes by IgG antibodies circulating in the bloodstream. It is, therefore, possible that linear peptides in the selected proteins may be inadequately suited to differentiate between these taxa. Given the incomplete histories and serology that was performed in a subset of our tested samples, additional work is needed to determine whether incomplete metadata contributed to this finding. It is also possible that quantifying the reactivity of peptides containing the range of amino acid variation that exists in natural strains could improve the overall reactivity of our peptide reagents. Additional laboratory experiments are being performed to calculate the specificity and sensitivity for our sets of peptides in a larger number of human serum samples from various clinical cohorts.

With these publicly accessible peptide array data, it could also be possible to perform the opposite analysis in a way that would search for regions that were recognized with reduced specificity and could therefore be useful to identify peptides that could indicate past infection by at least one of these viruses. Similarly, these data could be mined to identify linear peptides that could be used as antigens to generate an antibody response to such epitopes towards the development of additional “universal” monoclonal antibodies.

 The ELISA data indicate that this method could be a more resource- and time-efficient approach to PRNT. Although results against alternative characterization methods vary widely, additional criteria have been added to PRNT results to account for the high degree of cross-reactivity between ZIKV and DENV ^39^. The observed sensitivity and specificity values could potentially be improved through additional experimentation and optimization. Screening additional well-characterized samples with our ELISA method could shed additional light into a more accurate gauge of ZIKV seroprevalence and could guide public health decisions.

These data help to quantify the human humoral response to multiple mosquito-borne viruses and could be useful to identify, map, and/or design native or synthetic antigens that provide increased protection against natural infection by these viruses. Our data could also be relevant to the design of a mosquito-borne virus vaccine. However, care must be taken in designing such experiments to ensure that antibody-dependent enhancement does not increase the risk of adverse events following administration of the vaccine.

## Data availability

### Underlying data

Figshare: Peptide Arrays of Three Collections of Human Sera from Patients Infected with Mosquito-Borne Viruses. https://doi.org/10.6084/m9.figshare.c.4298600.v2 ^35^.

This project contains the following underlying data:

- 1 Metadata file for: Describing the characteristics about each patient from which serum was collected.
- 1 Metadata file for: Information on Peptide names, sequences, and identifiers included on Array.
- 1 Metadata file for: Metadata for Experimental Samples.
- 151 Data files containing quantitative data for the peptide arrays.

Data are available under the terms of the Creative Commons Zero “No rights reserved” data waiver (CC0 1.0 Public domain dedication).

## Software availability

The custom script used to parse the data files can be found at GitHub: https://github.com/bpickett/PeptideArray/tree/v0.9.

Archived source code at time of publication: https://doi.org/10.5281/zenodo.3518356 ^32^.

License: GNU General Public License v3.0.
