# Supplementary material for: Peptide arrays incubated with three collections of human sera from patients infected with mosquito-borne viruses
Source: F1000Res. 2020 Feb 28;8:1875. Originally published 2019 Nov 7. [Version 3] doi: 10.12688/f1000research.20981.3 (PMC7065662; doi:10.12688/f1000research.20981.3)
Supplement: Peer review history

# Peer review: Reviewer response for version 3

**DOI:** 10.5256/f1000research.24949.r60676

**Published:** 2020-03-10

**Authors:** Felix F. Loeffler

**Affiliations:** 1 Department of Biomolecular Systems, Max Planck Institute of Colloids and Interfaces, Potsdam, Germany

All of my previously raised questions and concerns have been sufficiently addressed. I believe that the manuscript can be accepted for indexing.

I confirm that I have read this submission and believe that I have an appropriate level of expertise to confirm that it is of an acceptable scientific standard.

# Peer review: Reviewer response for version 2

**DOI:** 10.5256/f1000research.24759.r60141

**Published:** 2020-02-18

**Authors:** Salvatore De-Simone

**Affiliations:** 1 Oswaldo Cruz Foundation, Rio de Janeiro, Brazil

The authors justified and revised the work accordingly.

I confirm that I have read this submission and believe that I have an appropriate level of expertise to confirm that it is of an acceptable scientific standard.

# Peer review: Reviewer response for version 1

**DOI:** 10.5256/f1000research.23090.r59099

**Published:** 2020-02-13

**Authors:** Felix F. Loeffler

**Affiliations:** 1 Department of Biomolecular Systems, Max Planck Institute of Colloids and Interfaces, Potsdam, Germany

In this manuscript entitled “Peptide arrays of three collections of human sera from patients infected with mosquito-borne viruses” by Pickett *et al*., the authors report the screening of patient sera using peptide microarrays for antibody interactions. The goal is to identify disease specific biomarkers, since “serological tests are often not reliable for diagnosis after seroconversion and convalescence due to cross-reactivity among flaviviruses”.

 Overall, the approach is sound and has very good potential and the authors did an impressive job in bioinformatic prediction and analysis. Yet, the results show that there is room for technological and methodological improvement.

 I believe that the data presentation can be improved. Thus, I suggest the following minor changes:

1. The title “Peptide arrays of … human sera…” is in my opinion misleading. “Human sera are analyzed/screened on/with peptide arrays” or “Arrays are incubated with human sera”.
2. It is somewhat difficult to understand the peptide selection, because there is just a reference to another publication. A brief 2-3 sentences description on the peptide selection process would help.
3. To judge the quality of the microarray data, it would be highly beneficial to have some kind of heat map of all array results together (raw and/or normalized median peptide staining intensity per patient). I found it somewhat unconventional to deposit a tsv result file on github. Only providing raw data makes a quick review and validation rather difficult. For an example, see Figure 2 in Weber, L.K., et al. (2017), *Eng. Life Sci.*, 17: 1078-1087 ^1^. Furthermore, I could not find a link between the raw data files and the corresponding peptide sequence. This needs more explanation and the authors should provide an additional file with the sequences of each position on the array.
4. In my opinion, the patient sample presentation has to be improved. The authors state that they only have incomplete patient history (“Given the incomplete histories and serology …additional work is needed to determine whether incomplete metadata contributed to this finding.”). Yet, in the section “Serum sources”, the authors give some ranges on patient age and days after onset of symptoms. In my opinion, a full patient information table (e.g. in supporting information) is essential (even though data might be missing), showing patient age, sex, known previous arbovirus infections (primary vs. secondary infection), days after onset of symptoms, etc. of all patients. From my experience, there can be a large difference in antibody response in patients 10 days vs. 30 days after onset of symptoms, so these patients should be categorized and analyzed as different groups. I believe that patient stratification is one of the keys to more statistically significant data.

The following general comments might be helpful to discuss the statistical significance of the results:

1. I do not fully agree with this general assumption: “Although reports showing antibodies against other viral proteins are detectable 12, the E and NS1 proteins are the primary targets of the humoral anti-flavivirus immune response in humans 13–15.” Although discussed before (citation 11), this approach ignores for example the highly specific serum biomarker from the NS2B protein in Zika. This approach might miss other similarly important peptide biomarkers. In addition, adding peptides from the nonstructural proteins might help to boost the overall sensitivity of the assay (yet, losing some specificity).
2. By choosing consensus sequences, the authors limited the peptide selection, which is valid and – with current array technology – necessary. However, this probably limits the sensitivity of the microarray results and the subsequent ELISA. Depending on the selected protein regions and patient origins (-> virus genotypes), there might be several important genotypic variants, which the authors would have neglected.

In addition, the array protocol seems not optimal and there are some inconsistencies in the methods section. Some general remarks, questions, corrections and suggestions regarding the array processing:

1. The raw data states that the scanner Innopsys Innoscan 1100AL and the Mapix software were used. Yet, the in the manuscript, it is stated: “…using a ProScanArray HT (Perkin Elmer) microarray scanner at 488 nm and 600 nm, and images were saved as high-resolution TIF files. Imagene® 6.1 microarray analysis software…”. Please correct. The scanner wavelengths were supposedly 488 nm and 600 nm. In the raw data files, it says 488 nm and 635 nm. Please correct. If possible, please also give the laser powers and gain (PMT) settings for the scans (according to the raw data: wavelengths 635, 488: "LaserPower=10.0, 5.0", "PMTGain=50, 50").
2. For the microarray experiments, the authors claim to have used the Alexa 488 labeled secondary antibody: Invitrogen, cat. #: A-11013, RRID: AB_2534080. This is an (H+L) chain specific antibody, so it will also bind to IgM antibodies (please include this information!)! The use of the Ab labeled with Alexa 488 is a bit odd, since the original data files state that the scanning of the main interaction was performed at 635 nm. Yet, the data of the 488 nm channel is not included in the raw data and, according to the protocol, it seems to me that this dye is only used for controls (see section “Peptide preparation and microarray printing”). Are you sure that you used this specific secondary antibody? If so, I would highly recommend changing to a 635 nm channel compatible secondary antibody, since the 488 nm channel gives much higher background and generally very strong autofluorescence.
3. In contrast, for the ELISAs, the authors apparently used a different secondary antibody, which is Fc specific, so it should only bind to IgG. Thus, the microarray data cannot be directly compared to the ELISA data.
4. The final steps of the array washing and drying protocol are in my opinion not optimal: “… another two times in deionized water and centrifuged to dry at 200 × g for 5 mins.” The pH of distilled water should be checked. Since deionized water is only weakly buffered (very low salt concentration), deionized water may have a low pH (especially observed in ddH2O). By washing the arrays with it, the lower pH may destroy interactions of weakly (or pH sensitive) binding antibodies. Instead, to remove salt residues from the array surface, you might want to use 1 mM Tris buffer in the future.
5. In addition, for future experiments, centrifuging arrays for drying is not optimal, since drying effects may cause artifacts (coffee ring drying effects, etc.). Instead, using a jet of air to quickly remove droplets from the surface causes much less artifacts.

Overall, the manuscript is well written and the bioinformatics seems sound. Yet, the authors can take some more care in presenting the data and should correct and/or explain the inconsistencies in the methods section (scanner, wavelengths, antibodies).

 Future analyses should focus on patient stratification and possibly more homogenous samples (different cohorts may be difficult to compare). This may significantly improve statistical outcome.

I confirm that I have read this submission and believe that I have an appropriate level of expertise to confirm that it is of an acceptable scientific standard, however I have significant reservations, as outlined above.

## References

[1] : Antibody fingerprints in lyme disease deciphered with high density peptide arrays. *Engineering in Life Sciences*.2017;17(10) : 10.1002/elsc.201700062 1078-1087 10.1002/elsc.201700062 PMC699947332624735

# Response

**Published:** 2020-02-25

**Authors:** Brett Pickett

**Affiliations:** Brigham Young University, USA

In this manuscript entitled “Peptide arrays of three collections of human sera from patients infected with mosquito-borne viruses” by Pickett *et al*., the authors report the screening of patient sera using peptide microarrays for antibody interactions. The goal is to identify disease specific biomarkers, since “serological tests are often not reliable for diagnosis after seroconversion and convalescence due to cross-reactivity among flaviviruses”.

 Overall, the approach is sound and has very good potential and the authors did an impressive job in bioinformatic prediction and analysis. Yet, the results show that there is room for technological and methodological improvement.

 I believe that the data presentation can be improved. Thus, I suggest the following minor changes:

 1)     The title “Peptide arrays of … human sera…” is in my opinion misleading. “Human sera are analyzed/screened on/with peptide arrays” or “Arrays are incubated with human sera”.

*Response: We thank the reviewer for their input on how to improve the title. We have changed the title to “Peptide arrays incubated with three collections of human sera from patients infected with mosquito-borne viruses”.*

 2)     It is somewhat difficult to understand the peptide selection, because there is just a reference to another publication. A brief 2-3 sentences description on the peptide selection process would help.

*Response: We agree with the reviewer and have incorporated a more detailed description of the peptide selection process in the last two paragraphs of the Introduction section, as well as the first paragraph of the Methods section in version 2 of the manuscript. We anticipate that these changes sufficiently clarify the peptide selection process that was informed by our previously published analysis.*

 3)     To judge the quality of the microarray data, it would be highly beneficial to have some kind of heat map of all array results together (raw and/or normalized median peptide staining intensity per patient). I found it somewhat unconventional to deposit a tsv result file on github. Only providing raw data makes a quick review and validation rather difficult. For an example, see Figure 2 in Weber, L.K., et al. (2017), *Eng. Life Sci.*, 17: 1078-1087 ^1^. Furthermore, I could not find a link between the raw data files and the corresponding peptide sequence. This needs more explanation and the authors should provide an additional file with the sequences of each position on the array.

*Response: We appreciate the reviewer making this useful suggestion. We have generated a heatmap to visually represent the normalized intensity ratios for each peptide predicted to be relevant for ZIKV. This new Figure 2 has been incorporated into the Results section and should adequately address this comment. We apologize that the link between peptide identifiers in the raw data files and the corresponding peptide sequences were not easy to find. The raw data files together with the metadata describing the peptide identifier on the array, the peptide name, and the peptide sequence are publicly available on Figshare (* https://doi.org/10.6084/m9.figshare.c.4298600.v2 *). We have improved the clarity of the file descriptions in the Underlying Data subsection within the Data Availability section of the manuscript. We believe that these adjustments should sufficiently enable readers to link the data files with the peptide sequence.*

 4)     In my opinion, the patient sample presentation has to be improved. The authors state that they only have incomplete patient history (“Given the incomplete histories and serology …additional work is needed to determine whether incomplete metadata contributed to this finding.”). Yet, in the section “Serum sources”, the authors give some ranges on patient age and days after onset of symptoms. In my opinion, a full patient information table (e.g. in supporting information) is essential (even though data might be missing), showing patient age, sex, known previous arbovirus infections (primary vs. secondary infection), days after onset of symptoms, etc. of all patients. From my experience, there can be a large difference in antibody response in patients 10 days vs. 30 days after onset of symptoms, so these patients should be categorized and analyzed as different groups. I believe that patient stratification is one of the keys to more statistically significant data.

*Response: We agree that providing these additional metadata will be useful in stratifying the data for future analyses and meta-analyses. We have added a new “Patient Metadata” file in the underlying data file collection on FigShare, have updated the Figshare DOI (* https://doi.org/10.6084/m9.figshare.c.4298600.v2 *), and have amended the file description text in the Underlying Data subsection within the Data Availability section of the manuscript.*

 The following general comments might be helpful to discuss the statistical significance of the results:

 1.     I do not fully agree with this general assumption: “Although reports showing antibodies against other viral proteins are detectable 12, the E and NS1 proteins are the primary targets of the humoral anti-flavivirus immune response in humans 13–15.” Although discussed before (citation 11), this approach ignores for example the highly specific serum biomarker from the NS2B protein in Zika. This approach might miss other similarly important peptide biomarkers. In addition, adding peptides from the nonstructural proteins might help to boost the overall sensitivity of the assay (yet, losing some specificity).

*Response: We thank the reviewer for pointing out this unclear phrasing. We have corrected the text in the Introduction section to address the usefulness of the nonstructural proteins as potential serological markers. We have also edited the second paragraph of the Discussion section to mention potential future experiments that would incorporate peptides from nonstructural regions.*

 2.     By choosing consensus sequences, the authors limited the peptide selection, which is valid and – with current array technology – necessary. However, this probably limits the sensitivity of the microarray results and the subsequent ELISA. Depending on the selected protein regions and patient origins (-> virus genotypes), there might be several important genotypic variants, which the authors would have neglected.

*Response: We appreciate the reviewer mentioning the concept that consensus sequence may be affected by strain- or type specific amino acid substitutions.* *We agree that the usefulness of this approach should be evaluated in future experiments as the capacity and capability of arrays continue to improve. We have added text to inform the readership of this potential weakness of our approach in the fourth paragraph of the Discussion section.*

 In addition, the array protocol seems not optimal and there are some inconsistencies in the methods section. Some general remarks, questions, corrections and suggestions regarding the array processing:

 1.     The raw data states that the scanner Innopsys Innoscan 1100AL and the Mapix software were used. Yet, the in the manuscript, it is stated: “…using a ProScanArray HT (Perkin Elmer) microarray scanner at 488 nm and 600 nm, and images were saved as high-resolution TIF files. Imagene® 6.1 microarray analysis software…”. Please correct. The scanner wavelengths were supposedly 488 nm and 600 nm. In the raw data files, it says 488 nm and 635 nm. Please correct. If possible, please also give the laser powers and gain (PMT) settings for the scans (according to the raw data: wavelengths 635, 488: "LaserPower=10.0, 5.0", "PMTGain=50, 50").

*Response: We thank the reviewer for finding these inadvertent errors in the text. We have changed the 600 nm wavelength in the manuscript to the correct value of 635 nm, which now matches what is reported in the raw data files. The instrument description in the raw data files is an artifact generated by the scanner software, while the instrument text in the manuscript is correct. We have also included the catalog information for the secondary antibodies, laser power information, and PMT gain in the “High-throughput screening and quantification of the characterized patient sera” subsection of the Methods section.*

 2.     For the microarray experiments, the authors claim to have used the Alexa 488 labeled secondary antibody: Invitrogen, cat. #: A-11013, RRID: AB_2534080. This is an (H+L) chain specific antibody, so it will also bind to IgM antibodies (please include this information!)! The use of the Ab labeled with Alexa 488 is a bit odd, since the original data files state that the scanning of the main interaction was performed at 635 nm. Yet, the data of the 488 nm channel is not included in the raw data and, according to the protocol, it seems to me that this dye is only used for controls (see section “Peptide preparation and microarray printing”). Are you sure that you used this specific secondary antibody? If so, I would highly recommend changing to a 635 nm channel compatible secondary antibody, since the 488 nm channel gives much higher background and generally very strong autofluorescence.

*Response: We appreciate the reviewer asking us to clarify the text surrounding the secondary antibodies that were used. Indeed, the secondary antibody used to detect serum antibodies bound to viral peptides were in the 635 nm channel, while the 488 nm channel was used solely for the secondary antibodies bound to the control peptides. We have added the correct catalog information for the secondary antibody used to detect reactivity to viral peptides in the “High-throughput screening and quantification of the characterized patient sera” subsection of the Methods section. We have added a new sentence in the third paragraph of the Discussion section to inform the readership that the H+L secondary antibodies that were used could also recognize bound IgM.*

 3.     In contrast, for the ELISAs, the authors apparently used a different secondary antibody, which is Fc specific, so it should only bind to IgG. Thus, the microarray data cannot be directly compared to the ELISA data.

*Response: The reviewer is correct. The microarray data were generated to narrow down the candidate viral peptides to a much smaller number that would be tractable to test with ELISAs. Changing the secondary antibody between the platforms does affect the ability to directly compare them, and some of the signal loss that was observed could be attributed to that change of reagents.*

 4.     The final steps of the array washing and drying protocol are in my opinion not optimal: “… another two times in deionized water and centrifuged to dry at 200 × g for 5 mins.” The pH of distilled water should be checked. Since deionized water is only weakly buffered (very low salt concentration), deionized water may have a low pH (especially observed in ddH2O). By washing the arrays with it, the lower pH may destroy interactions of weakly (or pH sensitive) binding antibodies. Instead, to remove salt residues from the array surface, you might want to use 1 mM Tris buffer in the future.

*Response: We appreciate the reviewer sharing this insight and we will take it into account when performing future experiments.*

 5.     In addition, for future experiments, centrifuging arrays for drying is not optimal, since drying effects may cause artifacts (coffee ring drying effects, etc.). Instead, using a jet of air to quickly remove droplets from the surface causes much less artifacts.

*Response: We thank the reviewer for providing such a useful recommendation to reduce the number of artifacts on the arrays that result from the drying process. We will incorporate this information in future experiments.*

 Overall, the manuscript is well written and the bioinformatics seems sound. Yet, the authors can take some more care in presenting the data and should correct and/or explain the inconsistencies in the methods section (scanner, wavelengths, antibodies).

*Response: We thank the reviewer for taking the time to review this manuscript so thoroughly and believe that the changes incorporated in the most recent version adequately address the inconsistencies that were found.*

 Future analyses should focus on patient stratification and possibly more homogenous samples (different cohorts may be difficult to compare). This may significantly improve statistical outcome.

*Response: We agree that stratifying the data in subsequent analyses could improve the findings. We look forward to more samples and data becoming available in the future.*

# Peer review: Reviewer response for version 1

**DOI:** 10.5256/f1000research.23090.r56293

**Published:** 2019-11-14

**Authors:** Salvatore De-Simone, David William Provance

**Affiliations:** 1 Oswaldo Cruz Foundation, Rio de Janeiro, Brazil

The authors have focused on an important biomedical problem – How to identify the virus responsible for an infection through serological diagnostic tests when many of the potential pathogens display a high level of sequence similarity? While the approach presented represents an excellent start to achieve their goals, a number of issues need to be addressed. They have correctly focused on short poly-amino acid sequences but need to go further and define the epitome of each virus. As an epitope defines the exact sequence of amino acids bound to the cleft of an immunoglobulin, it is the most precise restriction possible for differentiating similar pathogens. This would allow an *in silico* evaluation of the potential for cross-reactivity that could be confirmed by immunoassays. Furthermore, a peptide of 15 amino acids could limit reactivity with IgM, which is an important component to defining seroconversion. Another important point is that the panel of patient sera was fairly small. It is also unclear if each patient sample was screened for each of the pathogens in questions or only the suspected infection. It is very common for patients from endemic regions to suffer multiple infections without registering them. A small panel size also makes it difficult to perform *Receiver Operating Characteristics *analyses, which are absent from the study. In addition, a limited combination of peptides was tested rather than a checkerboard. This is all the more important as peptides often show the lowest sensitivity. Methodologically, another important point to be clarified is regarding the cut off value used in peptide immunoassays or the value used by the software to delimit the signal intensity of a positive test from a negative one.

We confirm that we have read this submission and believe that we have an appropriate level of expertise to state that we do not consider it to be of an acceptable scientific standard, for reasons outlined above.

# Response

**Published:** 2020-02-12

**Authors:** Brett Pickett

**Affiliations:** Brigham Young University, USA

*We thank the reviewer for sharing their insight about the importance of performing an epitome analysis. We agree that defining the epitome for these cross-reactive viruses would enable more sensitive downstream experimentation with immunoassays. At the time that our study was being performed a very low number of published linear B-cell epitopes existed in the literature. As such, we performed a bioinformatics analysis of existing sequence and structure data to identify linear regions in the three-dimensional protein structure that were both exposed on the protein surface and displayed a number of amino acid substitutions that significantly differed between each of the viral species that we evaluated. We apologize to the reviewer that this prior work was not adequately described in the manuscript. We have included additional text in the last two paragraphs of the Introduction section, as well as the first paragraph of the Methods section, to clarify the purpose and application of our previously published analysis.*

*We believe that the reviewer brings up a very useful point about the limited IgM reactivity of our peptides. It is true that applying these peptides to quantify IgM reactivity could expand the application of the 15-mer peptides as potential serodiagnostics. Although these experiments are important, the majority of the sera we evaluated in this study were collected after the point when IgM levels would produce a high enough signal for detection in these experiments. This is the primary reason that our current study focused on quantifying IgG reactivity.*

**

*We agree that the number of samples was limited for each of the three sera collections included in this study. The metadata about infection history for each of the samples varied by collection. The sera collections from biorepositories in the United States or Honduras were confirmed during the acute phase with various methods for the target virus. The non-Zika sera from Nicaragua were collected prior to the Zika outbreak, which minimized cross-reactivity. In contrast, the Zika-positive sera from Nicaragua that were included in our study were collected from pediatric patients who were enrolled in an ongoing clinical study and who had no prior infection with Flaviviruses. We have modified the text in the Serum Sources subsection within the Methods section to improve clarity.*

*We appreciate the reviewer pointing out that the ROC analyses were mistakenly left out. We have updated the manuscript with a new display item (Figure 2) that visualizes the results of the ROC analyses for each of the three individual sera collections, as well as for a combination of all collections. The relatively small number of sera was one of the primary reasons why we performed validation of our findings using ELISA.*

**

*The reviewer’s point describes one of the approaches that are commonly applied to identify important peptides. Our experimental design used a different approach that uses overlapping peptides. Briefly, sets of overlapping peptides, which span a IgG epitope, are included on the array to enable the quantification of antibody binding. Ideally, the signal increases over a set of overlapping peptides until it reaches the specific peptide with optimal antibody binding (and a corresponding peak in signal), followed by a decrease in binding over the remaining peptides. The signal then disappears once the antibody-specific epitope is no longer present on the peptide. Our design takes advantage of this approach at scale to facilitate better identification of peptides containing species-specific epitopes from the peptide array.*

*We thank the reviewer for pointing out this lack of clarity in our methodology. Briefly, normalized ELISA values above 2.5 were categorized as high-confidence, values between 1.5 and 2.5 as medium-confidence, and values below 1.5 as low-confidence. We have added text in the “ELISA Data Processing” subsection of the Methods section to clarify the previously published process.*
